# Gender based lung cancer risks for symptomatic coronary artery disease patients undergone cardiac CT

**DOI:** 10.1371/journal.pone.0265609

**Published:** 2022-04-11

**Authors:** Entesar Zawam Dalah, Abdulmunhem Obaideen, Sabaa Anam, Khalid Alzimami, Layal Khalid Jambi, David A. Bradley

**Affiliations:** 1 Department of Diagnostic Imaging, HQ Dubai Health Authority, Dubai, UAE; 2 Department of Medical Diagnostic Imaging, University of Sharjah, Sharjah, UAE Radiology; 3 University Hospital of Sharjah, Sharjah, UAE; 4 Department of Radiological Sciences, Applied Medical Sciences College, King Saud University, Riyadh, Saudi Arabia; 5 Centre for Nuclear and Radiation Physics, Department of Physics, University of Surrey, Guildford, Surrey, United Kingdom; 6 Centre for Biomedical Physics, School of Healthcare and Medical Sciences, Sunway University, Bandar Sunway, Selangor, Malaysia; University of Dundee, UNITED KINGDOM

## Abstract

We estimate the lifetime attributable risk (LAR) of lung cancer incidence in symptomatic Coronary Artery Disease (CAD) patients receiving enhanced Coronary Computed Tomography Angiography (CCTA) and the unenhanced Computed Tomography Calcium Scoring (CTCS) examination. Retrospective analysis has been made of CCTA and CTCS data collected for 87 confirmed CAD adult patients. Patient effective dose (E) and organ doses (ODs) were calculated using CT-EXPO. Statistical correlation and the differences between E and ODs in enhanced CCTA and unenhanced CTCS were calculated using the Pearson coefficient and Wilcoxon unpaired t-test. Following BEIR VII report guidance, organ-specific LARs for the cohort were estimated using the organ-equivalent dose-to-risk conversion factor for numbers of cases per 100,000 patients exposed to low doses of 0.1 Gy. Significant statistical difference (p<0.0001) is found between E obtained for CTCS and that of CCTA. The scan length was found to be greater in CCTA (17.5 ± 2.9 cm) compared to that for CTCS (15 ± 2 cm). More elevated values of dose were noted for the esophagus (4.2 ± 2.15 mSv) and thymus (9.6 ± 2.54 mSv) for both CTCS and CCTA. CTCS organ doses were lower than that of CCTA. Per 100,000 patients, female cumulative doses are seen to give rise to greater lung cancer LARs compared to that for males, albeit with risk varying significantly, noticeably greater for females, younger patients and combined CCTA and CTCS scans. While scan parameters and tube-modulation methods clearly contribute to patient dose, mAs offers by far the greater contribution.

## 1. Introduction

Patient radiation doses for cardiovascular computed tomography angiography (CCTA) are typically greater than that of other forms of radiological procedure [1]. While among the radiology fraternity this is very well understood, at the outset it nevertheless needs to be emphasized that in evaluation of coronary artery disease (CAD), CCTA has emerged as a highly effective non-invasive diagnostic imaging modality [2], albeit not replacing the invasive angiogram [3]. This is similarly true of the non-invasive computed tomography calcium score (CTCS) examination [4]. While both CTCS and CCTA are effective in evaluating CAD, the former shows greater sensitivity and specificity in identifying coronary artery stenosis for patients with 50% and 70% stenosis threshold [5]. Certainly, the clinical benefit of CCTA and CTCS examinations in assessing CAD far outweigh the risks from the radiation exposure. Thus said, the levels of ionizing radiation involved in these two procedures do carry a potential risk of radiation-induced cancer, albeit small. Accordingly, concerns have been raised in the scientific literature [2, 6–8]. It has been acknowledged that doses can be reduced by implementing diagnostic reference levels (DRLs) [9], leading to emphasized dose optimization and justification at an institutional level.

The lowest proposed radiation dose for which a reasonably reliable increase in risk of cancer can be projected is in the range 10 to 50 mSv for single exposures and 50 to 100 mSv for protracted exposures [10]. In this regard, it has also been suggested by Budoff et al. [11] that an effective dose of 10 mSv received in undergoing a CT study may yield an increased probability of inducing fatal cancer of approximately 1 in every 2000 cases. The Biological Effects of Ionizing Radiation VII [12] phase 2 report proposed gender- and age-specific lifetime risk estimates for the general population, presenting risk models for low dose low-LET radiation. They defined low dose as ≤ 0.1 Gy [13].

Effective dose (E), typically measured in mSv, are needed in evaluating patient risks and induced carcinogenesis caused by exposure to radiations [14–18]. In further regard to the BEIR VII phase- 2 report recommendations, for certain organs the organ dose measured in mSv can be used to estimate the lifetime attributable risks (LAR) arising from ionizing radiation exposure. In CT studies as referred to herein, it is essential to keep a record of patient doses, particularly CCTA and CTCS procedures [19]. Moreover, the Size-Specific Dose Estimate (SSDE) index provides quantification of the dose received by a patient during a scan, account being made for patient size. The SSDE is based on a series of conversion factors published by the American Association of Physicists in Medicine (AAPM) and is thus applied to the volume CT dose index (CTDIvol) [20].

Patient specific dose quantities such as E and organ dose (OD) measurements are important within the context of the clinical setup, seeking to ensure compliance with acceptable reference levels as well as consideration of justification and optimization of patient dose and image acquisition [21]. Given this, the main aim of present study was to estimate lung cancer associated risk for symptomatic CAD patients undergoing consecutive CTCS and CCTA examinations, use being made of the method proposed in the BEIR VII phase 2 report. Age- and gender-based lung cancer risk evaluation has also been explored. Furthermore, ODs, E and the accumulative E for symptomatic CAD patients subjected to CTCS and CCTA have all been estimated.

## 2. Materials and methods

### 2.1 Study design

This study was approved by the University of Sharjah and University Hospital of Sharjah Ethics Committees. Patient consent was not requested by the ethics committee since it’s a retrospective study. All patient related data were fully anonymized before getting access to the data. Data for a cohort of 87 symptomatic CAD patients were arbitrarily retrieved for retrospective study. The cohort selection criteria were confined to patients who had undergone two sequential scan protocols: enhanced CCTA and unenhanced CTCS. The CCTA and CTCS examinations were performed using a 128-slice CT scanner, specifically a Siemens Somatom Definition AS system (Siemens Medical Solutions, Forchheim, Germany). Automatic exposure control with fixed tube voltage of 120-kV and provision for tube current-time product (mAs) modulation based on body parts and thickness was applied in both examinations. Prospective ECG gating with craniocaudal scan direction was performed during a single inspiratory breath-hold to acquire CTCS.

### 2.2 CT-EXPO simulations

E and OD values were calculated using the Microsoft Excel application CT-EXPO v2.5 [22, 23], written in Visual Basic, providing for calculation of patient dose. E and ODs were estimated for scanner-specific and gender- and age-specific models, with tissue weighting (W_T_) factors from the revised International Commission on Radiological Protection publication 103 [24]. Furthermore, to estimate E and ODs, applied scanning acquisition parameters were used, including: scanner range data (slice positions) and input parameters such as tube voltage (in kV), electrical tube current (in mA), beam width (mm), table feed (mm) and slice thickness (mm). CTDIvol and the dose length-product (DLP) were measured in mGy and mGy.cm respectively.

### 2.3 LAR cancer estimation

E is the index of radiation risk to the general population from an examination calculated using conversion factors specific to each body part [16, 17]. The LAR for each patient was calculated using BEIR VII Phase2 reported age- and sex-specific models resulting from a single dose of 0.1 Gy [12]. Herein we estimated the lung cancer LAR, calculating the cumulative lung dose using CT-EXPO for each patient within the cohort, all having undergone sequential CCTA and CTCS examination. As an example, using the BEIR VII Phase-2 model [12] the lung cancer LAR for a 30-year-old male exposed to a dose of 0.1 Gy is estimated to be some 1 in 900.

### 2.4 Statistical analysis

Statistical analysis was performed using GraphPad Prism 7, V7.03, GraphPad Software, La Jolla, California, USA; Sigmaplot, V13.0, Systat Software, San Jose, CA, USA; and IBM SPSS, V22.0, SPSS Inc., Chicago, IL, USA. Statistical correlation and differences between E and ODs in enhanced CCTA and unenhanced CTCS were calculated using the Pearson coefficient and Wilcoxon unpaired t-test.

## 3. Results

### 3.1 Patients’ demographics and protocol

Tables 1 and 2 provide respective summaries for patient demographics and CCTA and CTCS scan details.

**Table 1:** Summarised patient demographics.

Patient Characteristics	Gender	Mean (SD), Range	Overall Mean (SD), Range
**Age (years)**	Female	59.0 (12.4), 35–85	56.1 (12.6), 33–85
	Male	52.3 (12.3), 33–77	
**PT weight (kg)**	Female	78.8 (16.4), 54.6–122.3	83.8 (19.7), 47.2–144
	Male	89.3 (21.0), 56.6–144	
**BMI (kg/m** ^**2**^ **)**	Female	32.6 (6.1), 24.3–48.3	31.2 (6.3), 19.6–51.6
	Male	30.2 (6.2), 19.6–51.6	

^SD^refers to standard deviation.

**Table 2:** Summary of CCTA and CTCS scan details and Acquisition Parameters (mAs and scan length).

Acquisition parameter	Cardiac Examination (n = 87)				Statistical difference between CTCS & CCTA
	CCTA		CTCS		
**Tube voltage**	120 kV		120 kV		Same
**Tube current**	400 mA		NA		NA
**Collimation width**	2×32×0.6 mm		2×32×0.6 mm		Same
**Rotation time**	330 msec		220 msec		Significant (p<0.0001)
**Table feed**	9.2 mm		34.5 mm		Significant (p<0.0001)
**Temporal resolution**	83 msec		NA		NA
**Slice thickness**	1.5 mm		3.0 mm		Significant (p<0.0001)
**mAs Mean (SD), Range**	**Female**	**Male**	**Female**	**Male**	NA
	146 (32.8), 69–205	131.9 (29.8), 66–185	76 (36), 34–195	66.3 (30.5), 28–199	
**mAs Mean (SD), Range**	138.5 (31.8), 66–205		70.9 (33.4), 28–199		Significant (p<0.0001)
**Scan length (cm) Mean (SD), Range**	**Female**	**Male**	**Female**	**Male**	NA
	17.1 (2.9), 13.3–26.8	17.4 (1.9), 13.8–23.2	14.8 (1.2), 34–195	15.3 (2.1), 10.7–18.2	
**Scan length (cm) Mean (SD), Range**	17.2 (2.4), 13–27		14.9 (1.8), 11–18		Significant (p<0.0001)

^NA^refer to not applicable; ^SD^refers to standard deviation.

### 3.2 Comparison between CCTA and CTCS based on E, mAs and scan length

**Fig 1:**
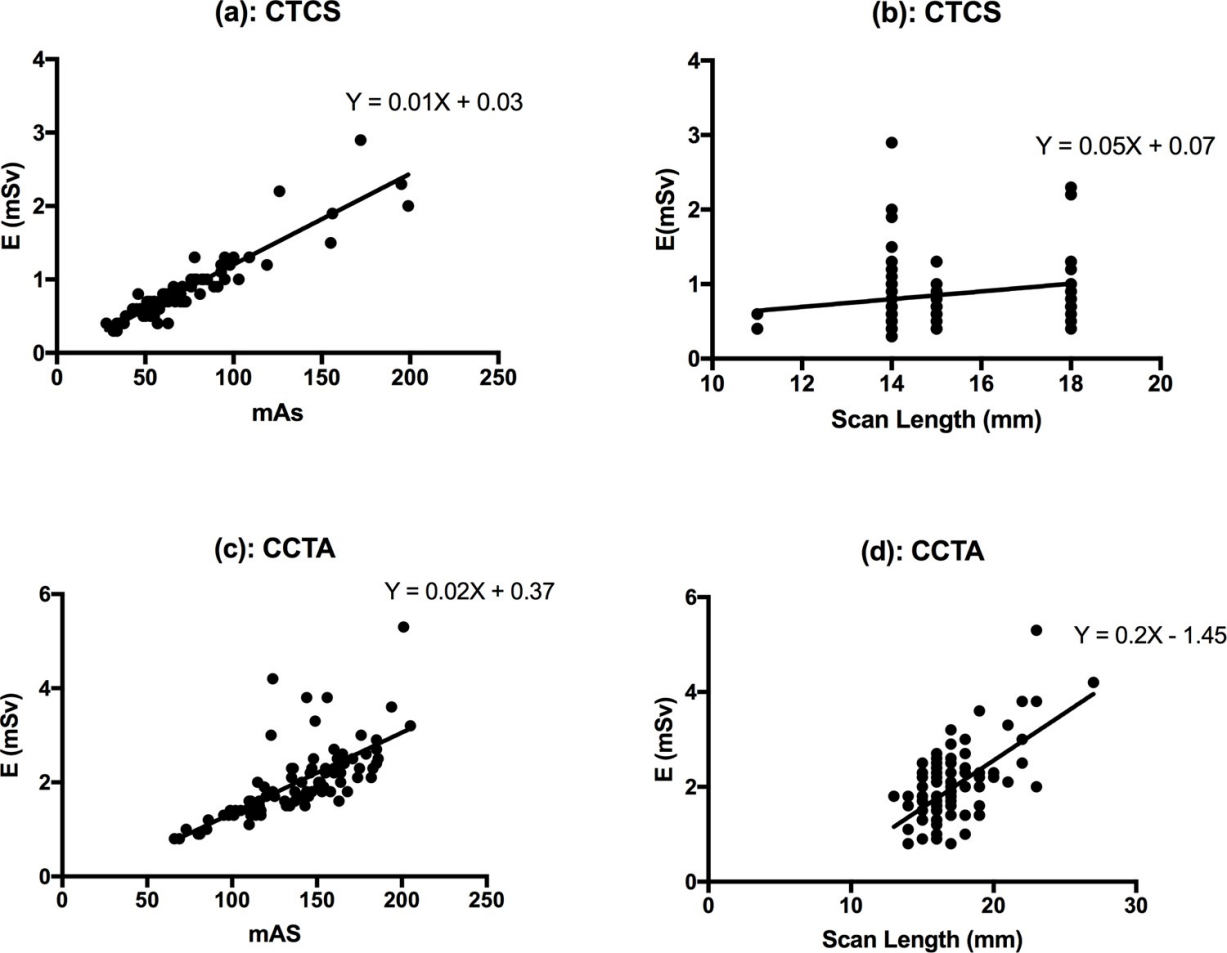
Scattered plot of: (a) effective doses (E) versus tube-current product (mAs) for CTCS; (b) effective doses (E) versus scan lengths (mm) for CTCS, (c) effective dose (E) versus tube-current product (mAs) for CCTA and;(d) effective doses (E) versus scan lengths (mm) for CCTA across all 87 patients.

In respect of CTCS for all 87 of the subject cohort (46 males and 41 females), Fig 1A shows the scatter plots, a linear regression relationship being observed between E and the mAs. Additionally, Fig 1B demonstrates the linear regression relationship between E and scan lengths used. Likewise, in respect of CCTA, Fig 1C indicates a linear regression relationship between E and mAs for all 87 of the cohort, with Fig 1D indicating the linear regression relationship between E and scan lengths used. Through use of the Pearson coefficient t-test, strong significant positive relationships have been found between E and mAs for CSCT and CCTA, at (r = 0.8095, p<0.0001) and (r = 0.9195, p<0.0001) respectively. Conversely, between E and scan lengths for CSCT there is an absence of any positive relationship (r = 0.2019, p = 00607) while for CCTA a moderate significant positive relationship is found (r = 0.6185, p<0.0001). Evident is that for almost every single patient a greater value of E is seen for CCTA compared to that for CTCS (Table 3). Similarly, the mAs is greater for CTCA than for CTCS, for all 87 patients, with females receiving greater mAs than males (Table 2). Larger scan lengths were used for the CCTA comparing against CTCS examinations. Table 2 also displays gender specific data for mAs and scan length.

**Table 3:** Chest-specific E comparison between CTCA and CTCS based on gender.

CCTA (n = 87)		CTCS (n = 87)	
E (mSv) Mean (SD), Range		E (mSv) Mean (SD), Range	
Male (n = 46)	Female (n = 41)	Male (n = 46)	Female (n = 41)
1.67 (0.43), 0.8–2.4	2.38 (0.9), 0.8–4.2	0.72 (0.32), 0.3–1.5	0.98 (0.53), 0.6–2.3

^SD^refers to standard deviation.

Using Wilcoxon paired t-tests, strong significant statistical difference (p<0.0001) was found between E for CCTA and CTCS. Likewise, strong significant statistical difference (p<0.0001) was found between the mAs values used for CCTA and CTCS. Finally, a strong significant statistical difference (p<0.0001) was seen in use of scan length for CCTA and CTCS.

### 3.3 Comparison between CCTA and CTCS based on SSDE and E

An evaluation of the SSDE has been performed for all 87 patients. Using the Pearson coefficient t-test, a strongly significant positive relationship between SSDE and E has been found at (r = 0.7057, p<0.0001) and (r = 0.9392, p<0.0001) for CCTA and CTCS respectively.

### 3.4 Organ doses

In making use of CT-EXPO, gender based descriptive organs dose analysis can be provided, as shown in Table 4. All six of the organs under study are seen to receive greater doses in use of CCTA compared to that in use of CTCS. Using the Wilcoxon paired t-test, the ODs for patients undergoing CCTA and CTCS were revealed to have a statistical significance (p<0.0001) for particular organs, as summarized in Table 4. In this study we used CT EXPO to obtain organs doses, with up to 30% associated error. Influencing factors that have acted upon the present and other studies in arriving at the estimated organs doses include detector configuration, protocols, pathology, and positioning.

**Table 4:** Organs Dose comparison between CTCA and CTCS based on gender, generated using CT EXPO, comparing the mean of CCTA to CTCS.

Organs	Gender	Organs Dose (mSv)			
		CCTA	CTCS	Cumulative	% Difference
		Mean (SD), Range	Mean (SD), Range		
**Oesophagus**	Female	9.6 (2.9), 3.2–16.6	4.0 (2.3), 1.6–12.6	13.6 (4.8), 4.8–25.6	82
	Male	9.5 (2.2), 4.7–13.6	4.4 (2.0), 2.1–12.8	13.9 (3.8), 7.4–24.6	73
**Lungs**	Female	4.8 (2.1), 1.4–11.4	1.8 (1.1), 0.7–6.2	6.6 (2.8), 2.1–15.4	91
	Male	5.6 (1.6), 2.6–9.1	2.2 (1.0), 0.9–5.8	7.8 (2.4), 3.6–12.4	87
**Liver**	Female	0.3 (0.2), 0.1–1.1	0.1 (0.1), 0.0–0.3	0.3 (0.2), 0.1–1.2	100
	Male	0.3 (0.1), 0.1–0.7	0.1 (0.1), 0.0–0.3	0.4 (0.2), 0.2–0.8	100
**Stomach**	Female	0.2 (0.1), 0.0–0.8	0.1 (0.0), 0.0–0.2	0.3 (0.2), 0.1–0.8	67
	Male	0.2 (0.1), 0.1–0.5	0.1 (0.0), 0.0–0.2	0.3 (0.1), 0.1–0.5	67
**Pancreas**	Female	0.3 (0.2), 0.1–1.0	0.1 (0.1), 0.0–0.3	0.4 (0.2), 0.1–1.1	100
	Male	0.3 (0.1), 0.1–0.8	0.1 (0.1), 0.0–0.3	0.4 (0.2), 0.2–0.8	100
**Heart**	Female	2.2 (1.8), 0.5–8.3	0.6 (0.5), 0.2–2.7	2.9 (2.0), 0.7–9.6	114
	Male	3.4 (1.5), 1.1–7.7	1.1 (0.6), 0.3–2.7	4.5 (1.9), 1.5–8.5	102

^SD^refers to standard deviation.

### 3.5 Lung cancer LARs

**Fig 2:**
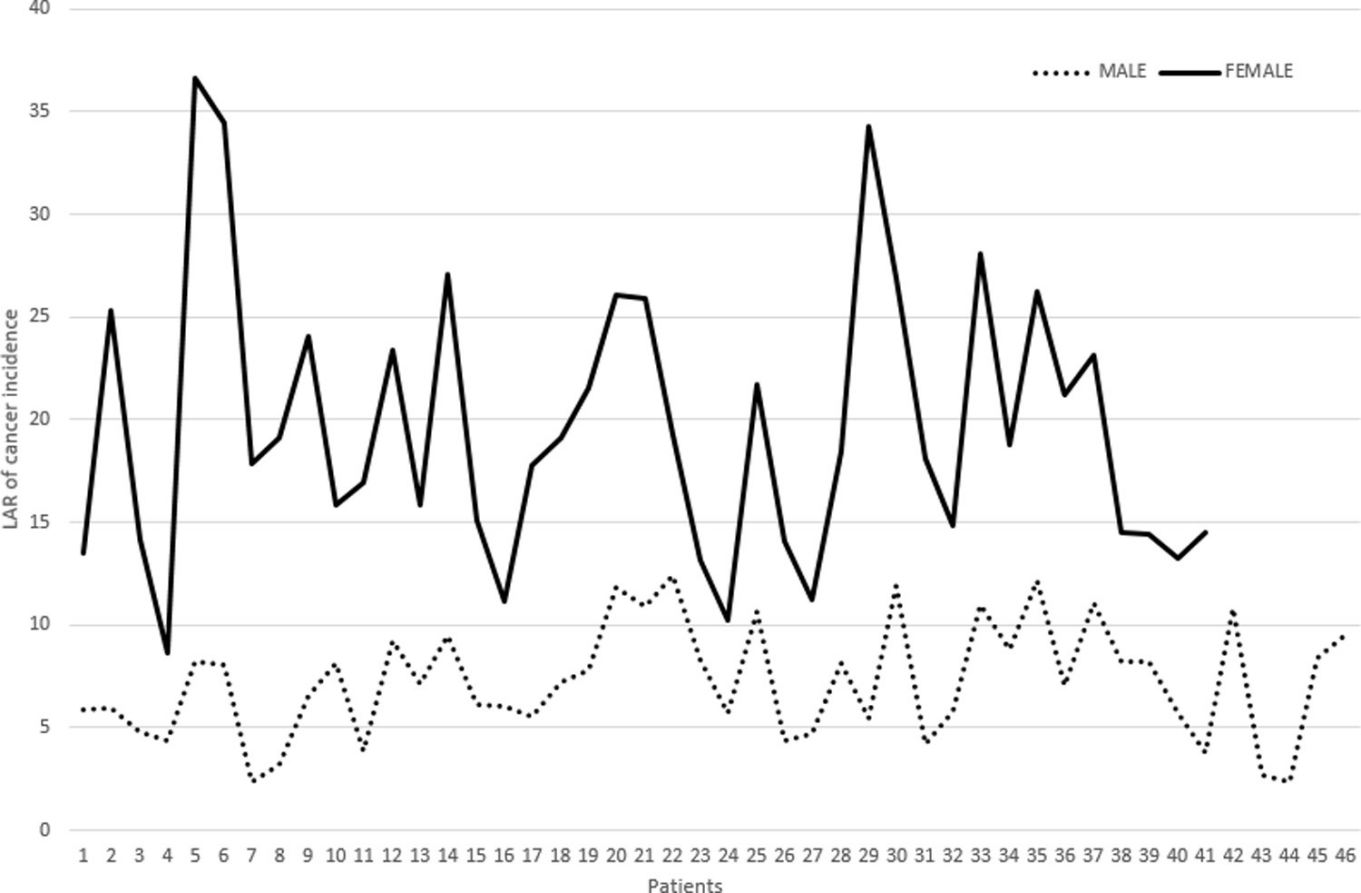
Comparison of radiation-induced lung cancer incidence between males and females per 100,000 patients, based on the cohort of 87 (46 males and 41 females) patients who underwent sequential CCTA and CTCS scans.

The risks were seen to be greatest for females in their 50s, decreasing as a function of age. The greatest LAR for lung cancer for a 40-year-old woman has been found to be 1 in 3500, for a 60-year-old woman 1 in 5000 and for a 70-year-old woman, 1 in 6500 per 100,000 patients. Fig 2. plots the cumulative lung cancer LAR for males and females subjected to sequential CTCA and CTCS scans.

The estimated lung cancer risks were noticeably lower in males than in females, being 1 in 8000 for a 50-year-old man, equivalent to the risk to an 80-year-old woman. It has also been demonstrated that the risks to lung cancer in men decreases as a function of age, 1 in 9000 for a 60-year-old man, 1 in 18,000 for a 70-year-old man and 1 in 43,000 for an 80-year-old man per 100,000 patients.

## 4. Discussion

In this study, for a cohort of 87 patients who received CCTA and CTCS scans in accord with scanning protocols, comparison has been made of E, ODs and lung cancer risk. Using conversion factors, CT scanner parameters such as CTDIvol and DLP can be translated into E and ODs, provided appropriate technical factors (scan length, kV) and patient characteristics (gender, age) are considered. From this, one can account for all exposed organs in a patient and compare the radiation received in CCTA examinations with that received in CTCS examinations.

A number of comparison studies have been summarized by Einstein et al [13], the work focusing on estimating E for cardiac CT examinations using Monte Carlo simulations. The mean E for CCTA ranged from 4 to 21.4 mSv, studies carried out using 64-slice scanners typically reporting greater doses due to the increased tube currents. The mean E for calcium scoring examinations was reported to range between 1 to 6.2 mSv, depending on the scanner and dose modulation methods used. Comparing the estimated mean E obtained in our study with those summarized by Einstein et al [13] one can conclude that the mean E obtained for CCTA and CTCS in the present 128-slice CT scanner study is considerably lower, by a factor of 0.1 and 0.2 respectively. While Einstein et al [13] determined the E using tissue weighting factor reported in ICRP 60 [25], the present work has relied on the updated tissue weighting factors reported in ICRP 103 [24]. Broad variations in mean E values have been reported for diagnostic CCTA examinations, examples including 11.4 mSv [26], 13.47 mSv [27], 18.1 mSv [28] and 19 mSv [2].

While E and ODs depend on tissue weighting factors, the conversion factors that have been used have relied on different estimation algorithms. These apart, DRLs strictly depend on machine output. Our institute DRLs for adult patients undergoing CTCS and CCTA have been published in a separate study [21], with 35 mGy. cm and 510 mGy.cm being the 50^th^ percentile of DLP for CTCS and CCTA respectively. Our DRLs have been shown to be considerably lower than that of a Swiss study of adult diagnostic reference levels, at 65 mGy.cm and 1222 mGy.cm for CTCS and CCTA respectively [29]. The lower CTCS DRLs of our institute are almost certainly due in good part to the use of a lower range of tube-current product (31–142, mAs) compared to that reported by Treier et al [29] (68–256, mAs), with the mAs range (58–205, mAs) used to carry out CCTA in our institute also being lower than that reported by Treier et al [29] (263–800, mAs). While the use of lower mAs values can be expected to result in noisier CT images, this should not be a problem given that diagnostically adequate image quality can still be obtained, a matter reflecting the implementation of image optimization in daily clinical routine. Present study has shown values of E and mAs to be greater in CCTA examinations compared to CTCS examinations.

Investigation has also been made of the scan acquisition parameters, with correlation analysis made using the Pearson correlation coefficient test. Significant (p<0.0001) statistical differences have been found between CCTA and CTCS for E, mAs and scan length. A stronger significant correlation was found between E and mAs for both CCTA (r = 0.9221, p<0.0001) and CTCS (r = 0.7385, p<0.0001) comparing to scan length. In particular, relying on automatic exposure control, also tube angulation and longitudinal tube-current modulation as well as tube potential modulation, one can be assured of greater patient dose optimization and minimized radiation exposure, provided that patient vertical and horizontal offsets are minimal. Studies have shown that an average of 2.2 cm horizontal mis-centering can yield an average dose increase of 23% [30], while a vertical displacement of 4 cm increases the dose by 67% [31]. The study also affirmed that mAs using angulation and longitudinal tube-current modulations would vary depending on patient body part composition and thickness, whereas the value of kV obtained in using tube-potential modulation would strictly depend on patient size. Similarly, clearly greater scan length has been observed in conduct of CCTA examinations compared to CTCS examinations, with means of 17.3cm and 15.1cm respectively.

For the CTCS cases of present study, use have been made of prospective ECG gating-based tube current modulation, with the scan performed by step-and-shoot acquisition, a situation in which the x-ray tube is energized only during the cardiac phase. Use of ECG-gating has been claimed to decrease radiation dose to the patient by approaching 45% [32] compared to CCTA examination carried out without ECG-gating [33]. Mean organ doses to the thyroid, esophagus, lungs, liver, stomach, heart, and other organs have been generated using CT-EXPO dosimetry. Our study shows sizeable differences in organ doses (ODs) between the two examinations. Organs that show relatively high doses are the esophagus (4.2 ± 2.15) mSv and thymus (9.6 ± 2.54) mSv for both CTCS and CCTA. Organ doses in CTCS were comparatively lower than that of CCTA. One major reason for the organ dose variations relates to differences in organ shape, volume, position inside the body, and the scan parameters summarized in Table 2. This includes the faster table feed and larger slice thickness that are associated with the CTCS examinations, resulting in shorter exposure times. Furthermore, scan length differences reflect in change in the field of view, a matter which could account in good part for the wide differences seen in values for some organ doses. At this point, one needs to highlight that CCTA is an enhanced examination procedure, meaning that a contrast medium is used, which could also contribute to greater dose [34]. One further factor is that an approximate error of up ± 30% can be expected when generating absolute organ doses using CT-EXPO [22].

The overall risk of a carcinogenic outcome from use of CCTA and CTCS can be estimated with the aid of E and ODs, with patient age and sex being explicitly considered [6]. In a study at the Medical University of South Carolina, a similar cancer risk analysis was carried out for 104 patients of median age 59 years who had undergone CCTA examinations. The study showed the organs receiving the greatest radiation doses were the lung (76 mGy), the estimated LAR for the radiation induced cancer risk being 0.12% [6]. In present study, we observed noticeable variation in age, sex and scan protocol for increasing risks of lung cancer. The estimated LAR for lung cancer varies significantly and has been noticeably greater for females, younger patients and combined CACT and CTCS scans. Table 3 demonstrates the gender-related situation for those undergoing CCTA and CTCS, with greater E received by females compared to males. For CCTA, for females the E mean (and range) is 2.38 (0.8–4.2) compared to 1.67 (0.8–2.4) for males. Similarly, for the CTCS procedure, for females within the cohort the E mean, and range is 0.98 (0.6–2.3) compared to that for males, at 0.72 (0.3–1.5). The greater E values for females is expected, linking directly to the associated higher mAs (Table 2) and evident greater body mass index, BMI, (Table 1), with the greater BMI giving rise to the need to increase mAs. The radio-sensitivity of the lung has been observed to decrease with age, as demonstrated in Table 4 for both males and females. Moreover, patients in the older age groups, are not only less radiosensitive but they are less likely to live long enough to develop lung cancer, accordingly with lower LARs than compared to that for younger patients. The LAR for lung cancer for female patients has also been found to be greater than for males in all age groups which may also be due to differences in radio-sensitivities as well as the amount of radiation exposure. Similar observations have been made in the study conducted by Einstein et al [13]. Recent studies like Karimizarchi & Chaparian [35] and Mahmoodi & Chaparian [36]; demonstrated similar findings in the sense that cancer induced risk is a variation of age and gender where younger females are at a higher risk.

The estimated risks in present study can provide researchers, radiologists and radiation safety officers with data that can be effective in assessing the risks versus benefits of CCTA and CTCS examinations. Further to be noted are that patients who smoke face a more elevated risk of developing lung cancer than others. Some 30% of the 1803 Life Span Study (LSS) subjects that were diagnosed with primary lung cancer were thought to be cases related to smoking, with only 7% associated with radiation exposure [37]. Perhaps and unsurprisingly, in a study of the joint effect of radiation and different levels of smoking on lung cancer incidence, heavy smokers were reported to be at significantly greater risk than low-to-moderate smokers [38, 39]. The relative risks per 1 Gy of radiation has been estimated to vary from 0.27 to 1.49, concerning not only histological types of lung cancer [38] but also other sites of respiratory tract cancers, including laryngeal, trachea, and mediastinum cancers [39]. Thus said, our approach in this study does have a number of limitations. One concerns the fact that in estimating the risk of lung cancer incidence, incorporation has not been made of patient size-corrected organ factors. Another is that others have commended the use of a linear non threshold model for radiation induced cancer [6]; it cannot be overlooked that low doses of radiation can accumulate over the long term, potentially resulting in radio-carcinogenesis. The risk estimates of present study have been made possible by use of the BEIR VII method which evaluates risks of low dose exposures ~ 100 mSv in a non-specific pathological population, not considering symptomatic coronary artery patients as herein. Furthermore, the commercially available software used to estimate organ doses are based on standardized uniform geometrical phantoms, hence not accounting for several factors that may have considerable impact of patient dose. Such factors include contrast material, patient habitus, organ size variations, and pathologies.

## 5. Conclusions

In conclusion, it is important to be aware of the risks to a patient from radiation dose, in present circumstances focusing on patients undergoing coronary CT angiography and CT calcium scoring examinations. In particular estimation of risk can better inform radiologists and CT operators in careful management of optimization of the imaging process. In present study, lung cancer risk has been found to be greatest for females. Dependent upon circumstances in clinical management of symptomatic cases, for females within the 50 to 60’s S1 Fig and S1 Table years age range consideration should be given of the option for alternative examinations utilizing non-ionizing radiation. With present study making detailed dose analysis, subsequent various dose reduction strategies can be considered towards lowering the radiation risks to patients undergoing CCTA and CTCS, including the use of different available automatic exposure control modulations. While it is clearly evident that the entire range of scan parameters contribute to patient dose, mAs remains by far the strongest dose contributor. It should always remain reasonable to consider dose reduction provided that the necessary medical information continues to be obtained.

## Supporting information

S1 Fig Comparison of radiation-induced lung cancer incidence between males and females per 100,000 patients, based on the cohort of 87 (46 males and 41 females) patients who underwent sequential CCTA and CTCS scans. (XLSX)

S1 Table Organs Dose comparison between CTCA and CTCS based on gender, generated using CT EXPO, comparing the mean of CCTA to CTCS. (XLSX)

## Abbreviations

CT: computed tomography
LAR: lifetime attributed risk
CAD: coronary artery disease
CCTA: coronary computed tomography angiography
CTCS: computed tomography calcium scoring
OD: organ dose
DRLs: diagnostic reference levels
BEIR VII: biological effects of ionizing radiation VII
E: effective dose
SSDE: size-specific dose estimate
AAPM: American Association of Physicists in Medicine
CTDIvol: volume CT dose index
